# Loss-of-function mutation in *Pcsk1* increases serum APOA1 level and LCAT activity in mice

**DOI:** 10.1186/s42826-021-00111-2

**Published:** 2022-01-07

**Authors:** Aleksandra Aljakna Khan, Nakyung Kim, Ron Korstanje, Seungbum Choi

**Affiliations:** 1 The Jackson Laboratory, 600 Main Street, Bar Harbor, ME 04609 USA; 2 Cerebrovascular Haematology-Immunology Priority Research Center, Medical Science Research Institute, Dongguk University Ilsan Hospital, Goyang, 10326 Republic of Korea

**Keywords:** Prohormone convertase, Lipoprotein, Cholesterol, High-density lipoprotein, Quantitative trait locus

## Abstract

**Background:**

The convertase subtilisin/kexin family 1 gene (PCSK1) has been associated in various human genetics studies with a wide spectrum of metabolic phenotypes, including early-onset obesity, hyperphagia, diabetes insipidus, and others. Despite the evident influence of PCSK1 on obesity and the known functions of other PCSKs in lipid metabolism, the role of PCSK1 specifically in lipid and cholesterol metabolism remains unclear. This study evaluated the effect of loss of PCSK1 function on high-density lipoprotein (HDL) metabolism in mice.

**Results:**

HDL cholesterol, apolipoprotein A1 (APOA1) levels in serum and liver, and the activities of two enzymes (lecithin-cholesterol acyltransferase, LCAT and phospholipid transfer protein, PLTP) were evaluated in 8-week-old mice with a non-synonymous single nucleotide mutation leading to an amino acid substitution in PCSK1, which results in a loss of protein’s function. Mutant mice had similar serum HDL cholesterol concentration but increased levels of serum total and mature APOA1, and LCAT activity in comparison to controls.

**Conclusions:**

This study presents the first evaluation of the role of PCSK1 in HDL metabolism using a loss-of-function mutant mouse model. Further investigations will be needed to determine the underlying molecular mechanism.

## Background

The convertase subtilisin/kexin family (PCSKs) are endopeptidases, which cleave immature proproteins (precursors) to generate mature functional proteins [1]. Their targets comprise various prohormones and proneuropeptides. Both rare and common mutations in PCSK1 (also known as prohormone convertase 1/3 or PC1/3) have been associated with obesity [2], and in various human genetic studies with a wide spectrum of metabolic phenotypes, including hyperphagia, intestinal malabsorption, gastrointestinal complications, diabetes insipidus, reactive hypoglycemia, and others [3]. PCSK1 cleaves its substrates at paired-basic amino acid residues [4]. More than 25 different targets of PCSK1 have been reported and most of them play an important role in metabolism [2, 3, 5]. Availability of several PCSK1 mouse models allowed investigating its role, expression, and tissue-specific substrates [5]. Until now, the role of PCSK1 has been studied primarily in brain, brainstem, pancreas, intestine, stomach, and more recently in immune cells [2, 5, 6].

Despite the evident influence of PCSK1 on adipose tissue and the known functions of other PCSKs in lipid metabolism, the role of PCSK1 specifically in lipid and cholesterol metabolism remains mostly unexplored [7]. Only a few association studies have reported a possible effect of PCSK1 on cholesterol. Two human PCSK1 mutations were linked with abnormal cholesterol levels in lipoprotein particles [8]. Lipoprotein particles are key vesicles in blood for transporting lipids and cholesterol to adipose tissue for storage and to other organs for utilization. One of those mutations was also associated with coronary artery disease in patients with type 2 diabetes [9]. In addition, the *Pcsk1* gene is located in the region that was previously identified as a quantitative trait locus influencing high-density lipoprotein cholesterol (HDL-C) in a mouse linkage analysis [10]. In this study, we evaluated the effect of loss-of-function in PCSK1 on HDL-C and factors influencing HDL metabolism such as apolipoprotein A1 (APOA1), lecithin-cholesterol acyltransferase (LCAT), and phospholipid transfer protein (PLTP) [11].

## Results

### Increased total serum APOA1 but similar HDL-C concentration in PCSK1^N222D^ mice

**Fig. 1:**
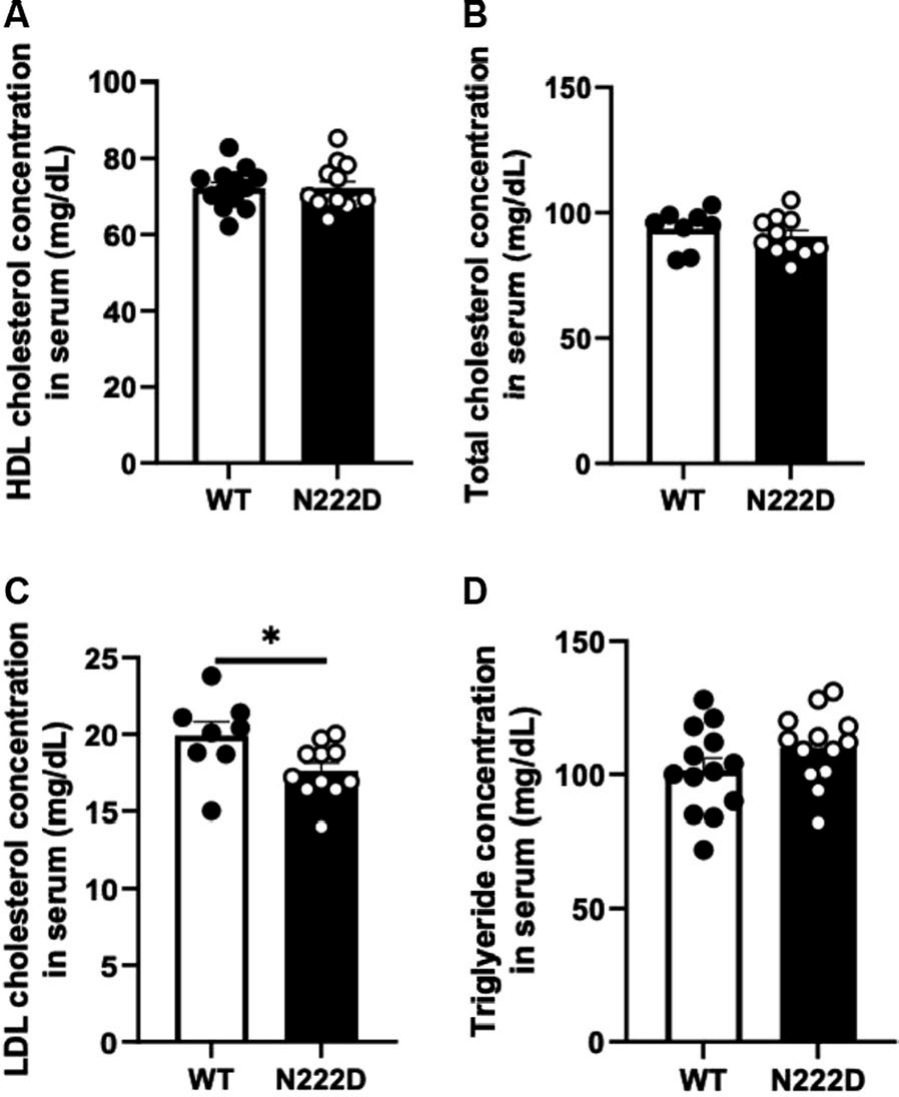
Similar serum HDL cholesterol concentration between PCSK1^WT^ and PCSK1^N222D^. **a** HDL cholesterol concentration (n = 13/genotype), **b** Total cholesterol concentration (n = 8–13/genotype), **c** LDL cholesterol concentration (n = 8–13/genotype), **d** Triglycerides concentration (n = 13/genotype). Serum was obtained from fasted 8-week-old PCSK1^WT^ and PCSK1^N222D^ males. Data represent the mean ± SEM from the number of animals of each group. *P < 0.05; HDL—high-density lipoprotein; LDL—low-density lipoprotein

**Fig. 2:**
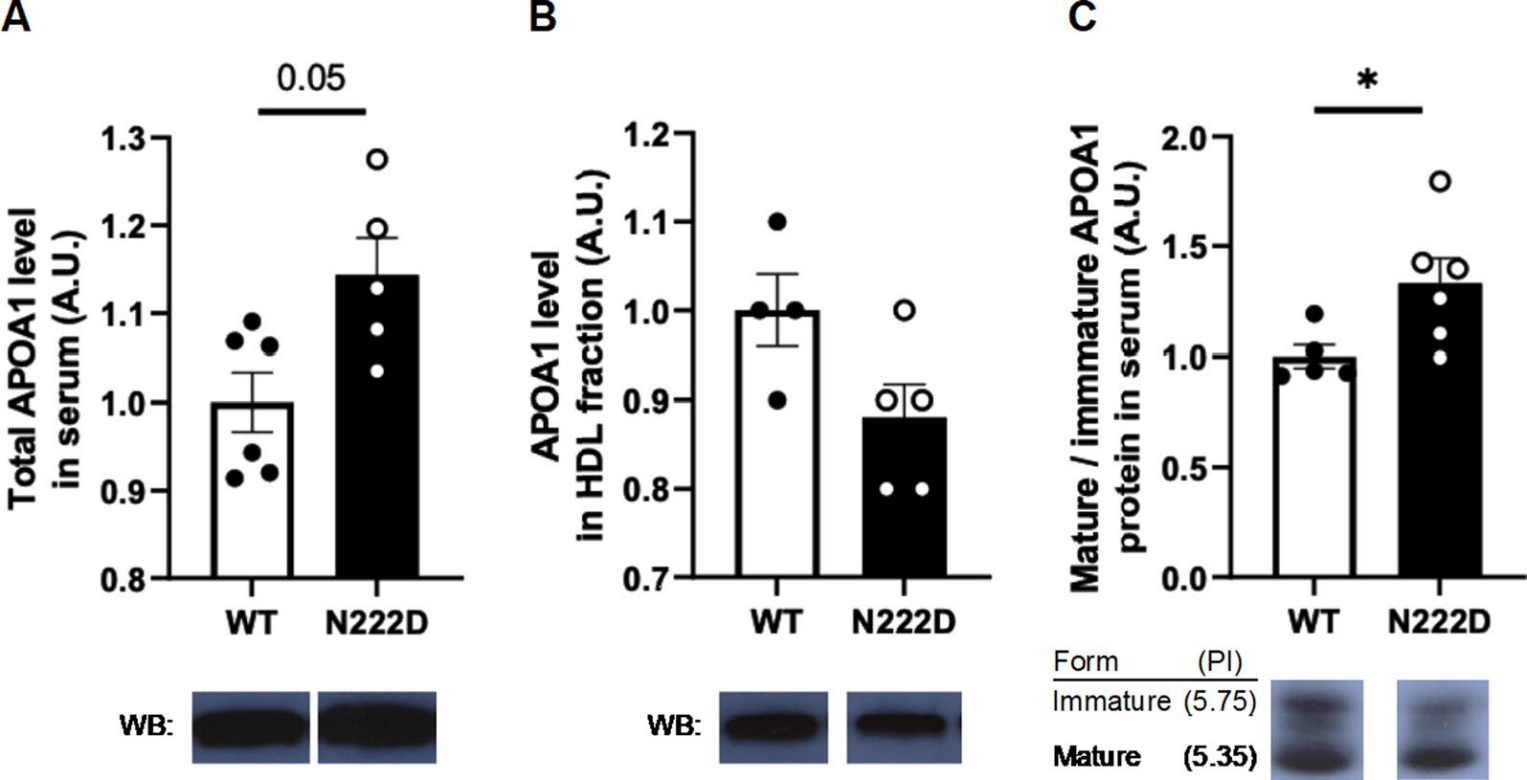
Increased mature lipid-free APOA1 level in PCSK1^N222D^ mice. **a** Top: increased serum total APOA1 level in PCSK1^N222D^ mice, (n = 5–6/genotype); bottom: western blot images of a representative band for APOA1 by genotype. **b** Top: similar APOA1 level in NHDS fraction, (n = 4–5/genotype); bottom: western blot images of a representative band for APOA1 by genotype. **c** Top: increased mature/immature APOA1 level (n = 5–6/genotype); bottom: IEF images of a representative band for immature and mature forms of APOA1 by genotype. Samples were obtained from fasted 8-week-old PCSK1^N222D^ and PCSK1^WT^ males. Data represent the mean ± SEM from the number of animals of each group. Equal serum volume was loaded into each well. WB —western blot, IEF —iso-electro focusing *P < 0.05

To test whether the amino acid substitution (N222D) in PCSK1 influences HDL metabolism, serum HDL-C concentrations and the total APOA1 level were compared between PCSK1^WT^ and PCSK1^N222D^ males (8-week-old, fed standard chow diet). Serum APOA1 is the major apolipoprotein in HDL. It plays an important role in HDL-C metabolism and is used to estimate the number of HDL particles. Although serum HDL-C concentrations were similar (WT: 72.22 ± 1.50 mg/dL vs. N222D: 72.32 ± 1.70 mg/dL; P = 0.84; n = 13/genotype) (Fig. 1a), the serum total APOA1 levels (HDL-bound plus lipid-free forms) were increased in PCSK1^N222D^ (WT: 1.00 ± 0.03 A.U. vs. N222D: 1.14 ± 0.04 A.U.; P = 0.05; n = 5–6/genotype) (Fig. 2a). Concentrations of serum total cholesterol, LDL-C, and triglycerides were also measured in PCSK1^N222D^ mice (Figs. 1 B-D). The concentrations of total cholesterol (WT: 93.5 ± 2.8 mg/dL vs. N222D: 90.5 ± 2.4; P = 0.47; n = 8 or 11/genotype, Fig. 2b) and triglyceride (WT: 101.6 ± 4.5 mg/dL vs. N222D: 110.1 ± 3.7 mg/dL; P = 0.17; n = 13/genotype, Fig. 2d) were similar between PCSK1^WT^ and PCSK1^N222D^. LDL-C concentration was 11.6% decreased in PCSK1^N222D^ (WT: 19.9 ± 0.9 mg/dL vs. N222D: 17.6 ± 0.5 mg/dL; P = 0.02; n = 8 or 11/genotype, Fig. 2c).

### Increased mature lipid-free APOA1 in PCSK1^N222D^ mice

**Fig. 3:**
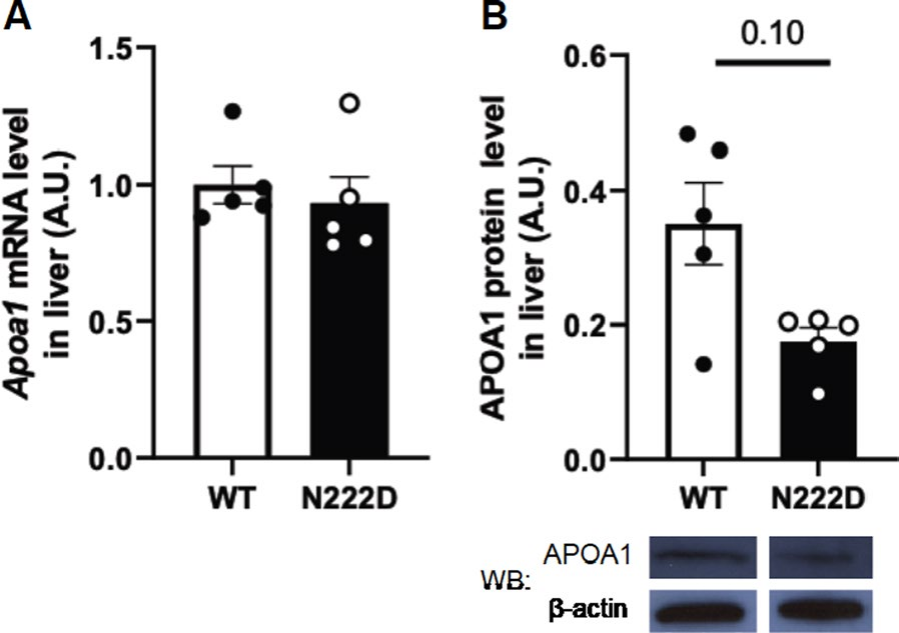
Comparison of *Apoa1* mRNA and APOA1 protein levels in liver of PCSK1^WT^ and PCSK1^N222D^ mice. **a** Similar liver *Apoa1* mRNA level, measured by qPCR, (n = 5/genotype), **b** Top: slightly reduced liver APOA1 protein levels (n = 5/genotype); bottom: western blot images of a representative band for APOA1 and β-actin by genotype. Tissue samples were obtained from fasted 8-week-old male mice. Equal amount of protein was loaded into each well. Relative level of APOA1 was calculated using the intensity of APOA1 protein band normalized by β-actin band. Data represent the mean ± SEM from the number of animals of each group. WB —western blot

APOA1 is synthesized primarily in the liver and is secreted into circulation as a precursor. The immature proprotein is cleaved into its mature form in blood, where it is present either as mature lipid-free or HDL-bound protein [12, 13]. In order to determine if lipid-free or HDL-bound APOA1 was increased in total serum, the APOA1 level was evaluated in non-HDL depleted serum (NHDS) fraction, which contains only HDL-bound APOA1. The levels of APOA1 in NHDS fraction were similar between PCSK1^WT^ and PCSK1^N222D^ (WT: 1.00 ± 0.04 A.U. vs. N222D: 0.88 ± 0.04 A.U.; P = 0.10; n = 4–5/genotype, Fig. 2b). The maturation of APOA1 in PCSK1^N222D^ males was evaluated using an iso-electric focusing (IEF) assay. The ratio of mature/immature APOA1 was increased by approximately 33% in PCSK1^N222D^ (WT: 1.00 ± 0.05 A.U. vs. N222D: 1.33 ± 0.12 A.U.; *P* < 0.03; n = 5–6/genotype, Fig. 2c). These results suggest an increase in the lipid-free mature APOA1 in circulation. Since APOA1 is produced primarily in liver, *Apoa1* mRNA and APOA1 protein levels were measured, and both mRNA and protein levels were not significantly different between PCSK1^WT^ and PCSK1^N222D^ (Fig. 3a, b).

### Moderately increased serum LCAT activity in PCSK1^N222D^ mice

**Fig. 4:**
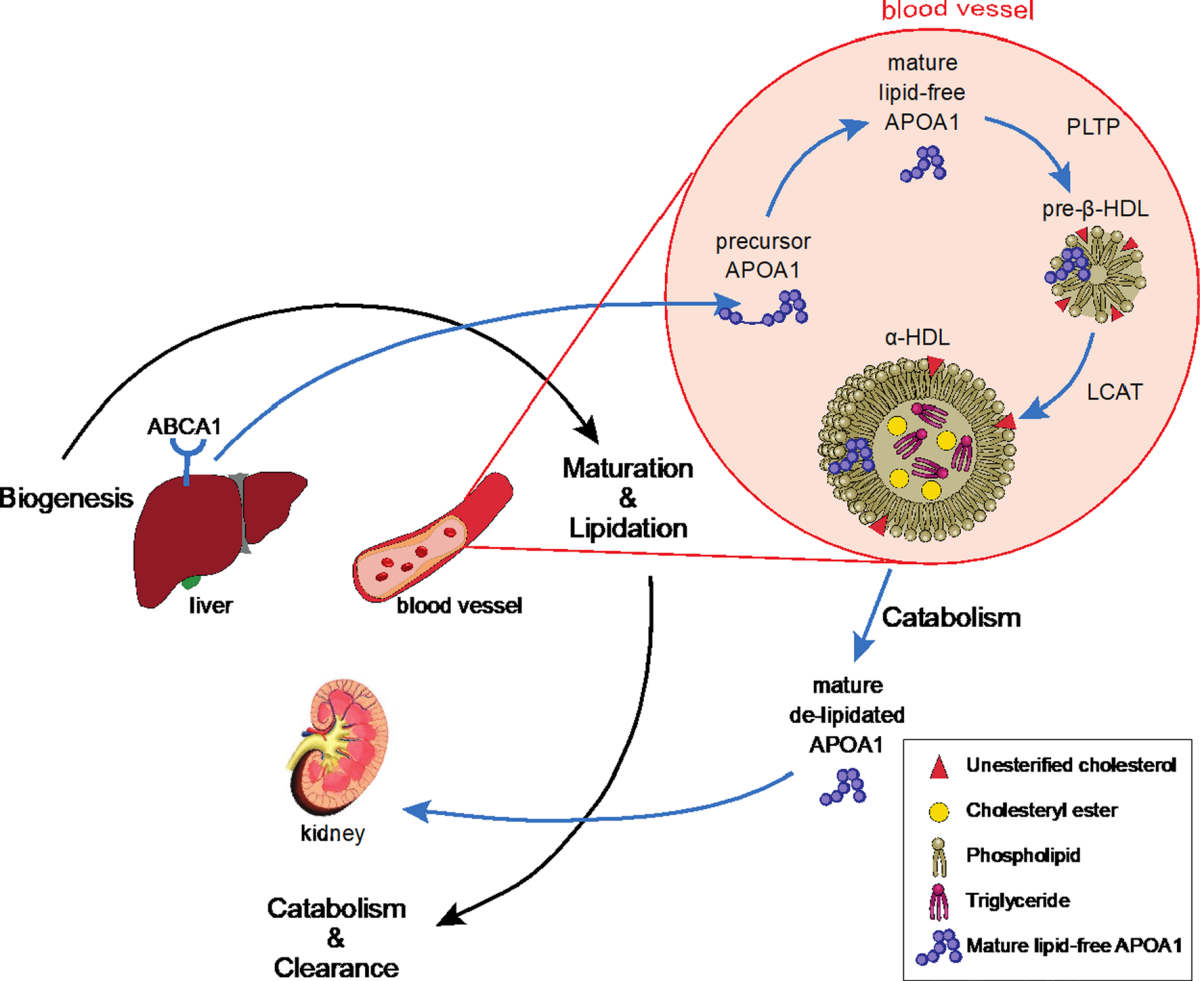
HDL biogenesis, remodeling and catabolism. APOA1 is predominantly produced in the liver and secreted into circulation as a lipid-free APOA1 precursor, which is subsequently converted to the mature APOA1 (APOA1 maturation). Initial APOA1 lipidation occurs by acquisition of phospholipids and unesterified cholesterol through phospholipid transfer protein (PLTP), leading to formation of lipid-poor HDL (also called pre-β-HDL). Further unesterified cholesterol conversion to cholesteryl ester happens through lecithin-cholesterol acyltransferase (LCAT), which turns the pre-β-HDL to a larger cholesterol-rich and triglyceride-containing HDL (also called α-HDL). APOA1 in the large α-HDL particles is released during HDL catabolism. The delipidated APOA1 is cleared through the kidney

**Fig. 5:**
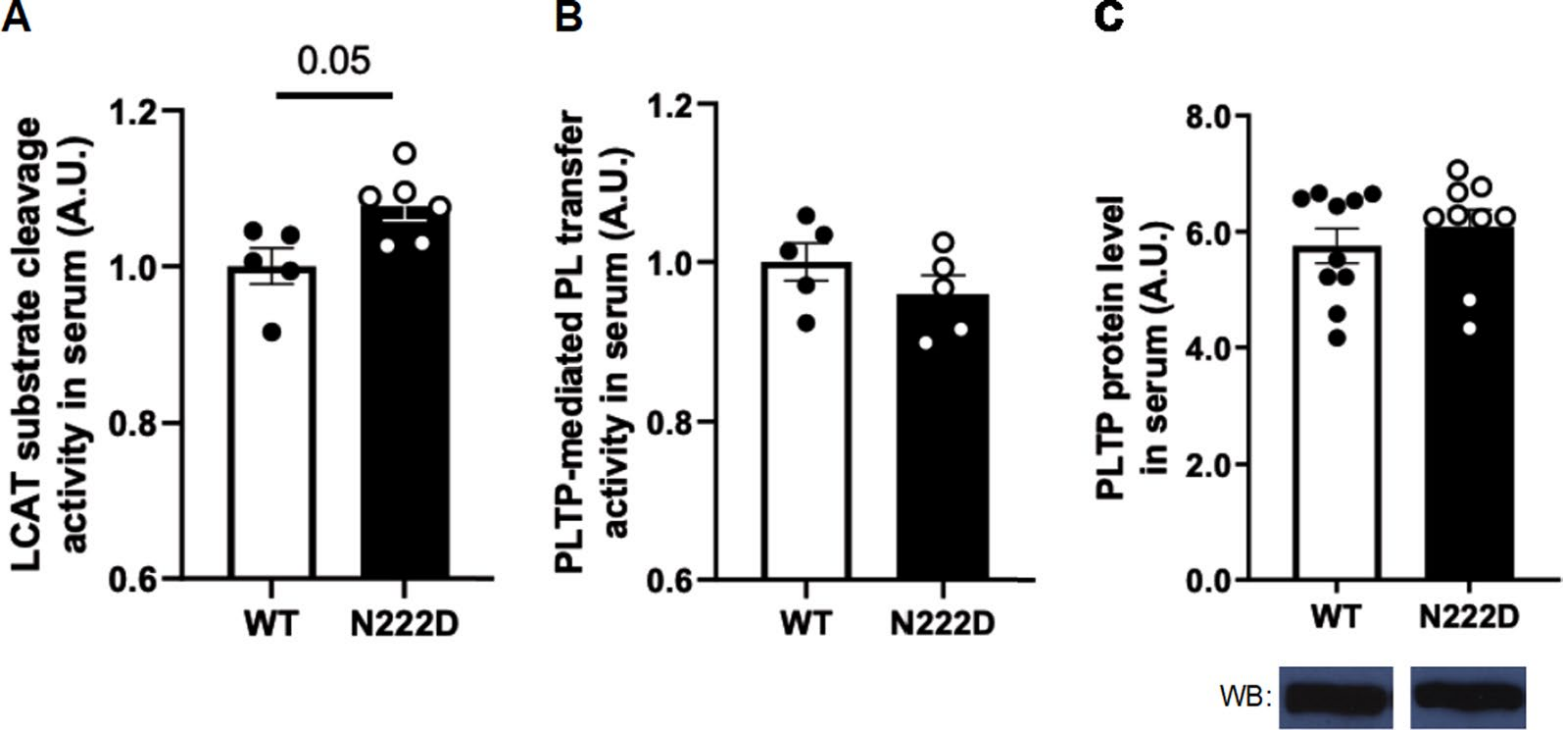
Serum LCAT substrate cleavage activity and PLTP activity/level in PCSK1^WT^ and PCSK1^N222D^ mice. **a** Moderately increased serum LCAT substrate cleavage activity in PCSK1^N222D^ mice (n = 5–6/genotype), **b** Similar PLTP-mediated phospholipid transfer activity (n = 5/genotype), **c** Top: similar serum PLTP protein level (n = 9–10/genotype); bottom: western blot images of a representative band for PLTP by genotype. Samples were obtained from fasted 8-week-old PCSK1^N222D^ and PCSK1^WT^ males. Equal amount of protein was loaded into each well

In circulation, the mature form of lipid-free APOA1 acquires phospholipid and cholesterol. This process is called APOA1 lipidation and initially results in the formation of nascent HDL particle (also known as pre-β HDL) (Fig. 4). The APOA1 in pre-β HDL activates LCAT, which subsequently converts free cholesterol into cholesteryl ester, thereby transforming pre-β HDL into larger spheroidal particles (α-HDL) [12, 14, 15]. The lipidation can be impaired by abnormal LCAT activity. Serum LCAT substrate cleavage activity was increased by approximately 8% in PCSK1^N222D^ mice (WT: 1.00 ± 0.02 A.U. vs. N222D: 1.08 ± 0.02 A.U.; P = 0.05; n = 5–6/genotype, Fig. 5a). Trend towards an increased cholesteryl esterification (the ratio of cholesteryl ester to free cholesterol) was also observed in the PCSK1^N222D^ relative to PCSK1^WT^ (WT: 2.58 ± 0.44 A.U. vs. N222D: 3.18 ± 0.62 A.U.; P = 0.08; n = 6/genotype, data not shown), and this was a similar trend as the LCAT substrate cleavage activity.

### Similar serum PLTP activity between PCSK1^N222D^ and PCSK1^WT^ mice

Acquisition of phospholipids is another important step in APOA1 lipidation and formation of HDL particle and PLTP is an enzyme that was shown to be implicated in this process by transferring phospholipids from triglyceride-rich lipoproteins to HDL or between different HDL subpopulations (Fig. 4) [12, 15–17]. Serum PLTP activity was found similar between PCSK1^WT^ and PCSK1^N222D^ mice (WT: 1.00 ± 0.02 A.U. vs. N222D: 0.95 ± 0.02 A.U.; P = 0.22; n = 5/genotype, Fig. 5b). Serum PLTP protein level was also similar in PCSK1^N222D^ relative to PCSK1^WT^ (WT: 1.00 ± 0.02 A.U. vs. N222D: 1.08 ± 0.03 A.U.; P = 0.55; n = 9–10/genotype, Fig. 5c).

## Discussion

Although several human PCSK1 mutations have been associated with obesity and two human single nucleotide variants (SNVs) were linked with abnormal lipids in lipoprotein particles, the effect of PCSK1 on lipid and cholesterol metabolism remained largely unclear. This study presents the first evaluation of HDL metabolism in the PCSK1^N222D^ mice [5, 18]. Our data shows that a loss-of-function mutation in PCSK1 leads to increased mature lipid-free APOA1 levels and LCAT activity without increasing HDL-C. We observed a different expression pattern of APOA1 protein in liver and serum: similar APOA1 protein level in the liver versus increased APOA1 protein level in the serum of the PCSK1^N222D^ mice relative to PCSK1^WT^ mice. Similar hepatic APOA1 (accompanied with similar *Apoa1* mRNA level) suggests that hepatic production of APOA1 (the first stage of HDL biogenesis) is unaffected by the mutation. These results may be suggestive of (1) an effort to increase lipidation of HDL particles, or (2) the result of increased HDL delipidation/catabolism (the last stage of HDL biogenesis), but this needs to be supported by additional experiments.

Further research on several additional aspects of HDL metabolism would help elucidating a more precise cause for the observed phenotype in PCSK1^N222D^ mice. Firstly, cholesterol loading into HDL depends on a proper function of several membrane proteins such as ATP-binding cassette subfamily member 1 transporters (ABCA1, a cell membrane transporter involved in cholesterol export from the cells), ATP-binding cassette subfamily G member 1 (ABCG1), and scavenger receptor class B member 1 (SRB1) [12, 19]. Whether the function of any of these proteins is impaired in PCSK1^N222D^ mice still needs to be evaluated. Secondly, elevated lipid-free APOA1 levels in circulation can be a result of increased catabolism of HDL particles. Such delipidated APOA1 is cleared preferentially by kidney and excreted via urine [19, 20]. Increased catabolism of HDL particles without concurrent increase in clearance by kidney remains a possible explanation for increased serum APOA1 levels and should be evaluated experimentally. Furthermore, HDL metabolism is affected by the concentration of different HDL subpopulations and their compositions (e.g. levels of serum amyloid A in HDL) [12]. These aspects have not yet to be investigated in this mouse model. Lastly, the oxidation of a specific amino acid on APOA1 was recently reported as important modification for HDL dysfunction in plasma [12]. This modification also remains to be tested. Interestingly, infusion of recombinant APOA1 and LCAT is being considered and investigated as a potential therapeutic strategy [12]. Our data shows that at least under some conditions HDL-C can remain low despite increased lipid-free APOA1 and increased LCAT activity.

Understanding HDL metabolism was initially of high interest because HDL-C is inversely associated with cardiovascular disease [12]. Historically, HDL-C was considered anti-atherogenic because of its role in reverse cholesterol transport (a process that mediates cholesterol efflux from macrophages in atherosclerotic lesions). The research over the last decades demonstrated that HDL has also anti-platelet, anti-oxidative, anti-inflammatory, anti-apoptotic and vasodilatory functions. Interestingly, knockdown of *Pcsk1* in macrophages was recently demonstrated to increase secretion of proinflammatory factors [21]. Future research investigating macrophage-related inflammatory phenotype the PCSK1^N222D^ mice would be of interest.

Unlike previously published human association studies, our data shows that loss-of-function mutation in PCSK1^N222D^ does not affect HDL-C in mice (although it does lead to lower LDL-C). The observed difference might be related to the location of the SNVs and their effect on gene expression or the ultimate function of the protein. In the mouse model used in this study, the mutation is in the protein coding region (A > G in codon 222, catalytic domain), which leads to a loss of protein’s function. The two SNVs that were previously associated with cholesterol and triglycerides in human studies are rs3811951 (intron 2, A > G) and rs155971 (intron 6, C > T). Both of the SNVs are intronic and their effect on PCSK1 expression is yet to be investigated. Until recently, the research was focused primarily on non-synonymous SNVs in the protein-coding regions. Interestingly, many of the newly discovered disease-associated SNVs are located in the non-coding region (intergenic or intronic) [22]. Our understanding of their role is only beginning to unravel [23]. Some of the intronic variants have been shown to impact splicing by interfering with splice site recognition or affecting the binding of RNA-binding proteins [24].

Aside from human studies, *Pcsk1* was also an HDL-C candidate gene in a mouse quantitative trait locus in a cross between BALB/cJ and B6.C-H25c (a congenic strain carrying the BALB/c Apoa2 allele —chromosome 1) [10]. The causal single nucleotide polymorphism (SNP) is yet to be identified. Nonetheless, several *Pcsk1* SNPs between BALB/cJ and B6 have been annotated in The Jackson Laboratory Mouse dbSNP data-base [25], including rs8274592 (a coding but synonymous SNP), rs8267200 (in the 3’ untranslated region (UTR) of messenger RNA), and various predicted intronic SNPs. The 3’-UTR can contain binding sites for regulatory proteins and microRNAs that control post-transcriptional expression of the gene or localization of the protein [26, 27]. Lastly, two different splice isoforms of *Pcsk1* have been annotated in the Ensemble. The difference between these two isoforms remains unexplored and the SNVs and SNPs might have a unique effect on each isoform.

Our study provided characterization of HDL metabolism in PCSK^N222D^ mouse model. Despite the increased lipid-free mature APOA1 and moderately increased LCAT activity, HDL-C levels remained unchanged in mice with a dysfunctional PCSK1 (caused by a SNP in the region coding for catalytic domain).

## Conclusions

Overall, this study provides the first preclinical evidence for a novel and unexpected function of PCSK1 in cholesterol metabolism. We suggest further studies to reveal underlying molecular mechanisms by which PCSK1 modulates serum APOA1 concentration and LCAT function without affecting serum HDL cholesterol concentration.

## Methods

### Animals, housing, and diet

Animal studies were approved by The Jackson Laboratory's Institutional Animal Care and Use Committee. All animals were housed at The Jackson Laboratory, which is approved by the American Association for Accreditation of Laboratory Animal Care and maintained in a pathogen-free and climate-controlled facility with a 12-h light/dark cycle and fed ad libitum (except for the times of blood collection as described below) a normal chow diet containing 6% fat (5K52 LabDiet®, PMI Nutrition International, St. Louis, MO, USA) [7]. The PCSK1 loss-of-function mutant mouse model (C57BL/6 J-Pcsk1^N222D^/J (JAX® #006,699), referred to as PCSK1^N222D^ throughout the rest of the article) contains an adenine-to-guanine transition, which leads to an amino acid substitution in the catalytic domain from asparagine (N) to aspartate (D) at codon 222. Only 8-week-old males (20 ~ 25 g) were utilized in the present study.

### Blood collection and serum preparation

Mice were fasted from 07:00 am to 11:00 am and then retro-orbitally bled; 100–150 µl of blood was collected in a 1.5 ml tube for serum. Serum was isolated by centrifugation at 15,000 rpm for 5 min at room temperature within 2 h of the bleed. Collected supernatant was stored at − 20 °C until needed for experimental measurements. Interventional studies involving animals or humans, and other studies that require ethical approval, must list the authority that provided approval and the corresponding ethical approval code.

### Analysis of HDL cholesterol, total cholesterol, and triglyceride

The HDL-C concentration was measured using an HDLD reagent kit (Beckman Coulter Inc., Palo Alto, CA, USA) on a Beckman Synchron DXC (Beckman Coulter Inc., Palo Alto, CA, USA). The total cholesterol concentration was measured using a CHOL reagent kit (Beckman Coulter Inc., Palo Alto, CA). Low-density lipoprotein cholesterol (LDL-C) concentration was estimated by subtracting HDL-C concentration from the total cholesterol. Triglycerides concentration was measured using a triglyceride reagent kit (Beckman Coulter Inc., Palo Alto, CA). The methods used for measuring HDL-C, total cholesterol, and triglycerides concentrations were validated in mice and were used in our previous publications [7]. Measurement for total cholesterol and LDL-C in certain serum samples were unsuccessful for technical reasons. These measurements were excluded from statistical analyses. Thus, while HDL-C comparison included a total of 13 samples per genotype, the number of animals per genotype in the comparison of total cholesterol and LDL-C is slightly lower.

### Western blot (WB) for APOA1

APOA1 was measured in non-HDL depleted serum (NHDS) and liver. NHDS was collected as previously described [7]. Briefly, the NHDS was prepared by separating the sample into 2 parts: 1) a precipitated fraction with all of the non-HDL lipoprotein particles (namely, VLDL, LDL, and IDL) as well as lipid-free APOA1, and 2) supernatant containing HDL and HDL-bound APOA1 (the NHDS fraction). This precipitation was done by dextran sulfate and magnesium chloride precipitation method. Each sample was mixed with one-tenth volume of the chemical precipitation reagent containing dextran sulfate (10 g/L) and magnesium chloride (500 mM), which lead to precipitation of non-HDL particles [28].

To compare the APOA1 in NHDS by WB [7], the samples were diluted in T-PER agent (Roche, Indianapolis, IN, USA) and a protease inhibitors cocktail tablet was added (Roche, Indianapolis, IN, USA). Equal volumes (μl) of diluted NHDS samples were electrophoresed using sodium dodecyl sulphate–polyacrylamide gel electrophoresis (SDS-PAGE) and transferred to a membrane, which was then probed using primary polyclonal rabbit APOA1 antibody (ab20453, 1/1,000, Abcam, Cambridge, MA, USA) and secondary antibody for anti-rabbit IgG (7074S, 1/5,000, HRP-linked secondary, Cell Signaling Technology Inc., Danvers, MA, USA). The APOA1 band intensity was normalized using total protein concentration (μg/μl), which was determined by a Bradford assay (Sigma Life Sciences, St. Louis, MO, USA).

To evaluate APOA1 in liver, the tissues were ground frozen on dry ice and diluted in T-PER agent (Roche, Indianapolis, IN, USA). Then, a protease inhibitors cocktail tablet was added (Roche, Indianapolis, IN, USA). Total protein concentration (μg/μl), which was determined using a Bradford assay (Sigma Life Sciences, St. Louis, MO, USA). Equal amount of protein (μg) was electrophoresed using SDS-PAGE and probed using primary polyclonal rabbit APOA1 antibody (ab20453, Abcam, Cambridge, MA, USA; 1/1,000) and primary polyclonal rabbit β-actin antibody as control (ab8227, 1/25,000, Abcam, Cambridge, MA, USA). The APOA1 band intensity was normalized by β-actin protein band intensity. For both serum and liver, protein band intensities were measured using ImageJ (National Institute of Health, Bethesda, WD, USA).

### Iso-electro focusing (IEF) for mature and immature APOA1

Serum (50 μl) was combined with protein extraction reagent type IV containing 37.5 μl of 200 mM tributylphosphine solution (Sigma-Aldrich, St. Louis, MO) and 15 μl of 1 M acrylamide. The resulting mixture was incubated at room temperature for 1.5 h and centrifuged at 15,000 rpm for 10 min. Collected supernatant was mixed with 9 volumes of acetone, incubated for 1 h at room temperature, and centrifuged at 4,000 rpm for 30 min. The obtained precipitated proteins were dissolved in 200 μl of protein extraction reagent type IV and the total protein concentration was measured by Bradford assay.

The separation of mature and immature APOA1 was done using IEF method according to the manufacturer’s instructions. Briefly, serum proteins were separated using gel electrophoresis technique in two consecutive steps: 1) the 1st dimension separation by molecular weights using SDS-PAGE, and 2) the 2nd dimension separation by isoelectric points using IEF gel: equal amount of total protein (15 μg) in IEF pH 3–7 sample buffer was loaded and run in Novex® pH 3–10 IEF gel (Invitrogen, Grand Is-land, NY, USA) at 100 V, 200 V, and 500 V for 1 h at each voltage. Then, the two forms of APOA1 were visualized by WB using APOA1 antibody as described above, and their band intensities were measured using ImageJ. Relative levels were calculated by dividing the protein band intensities of mature APOA1 by immature APOA1.

### Lecithin-cholesterol acyltransferase (LCAT) activity

LCAT substrate reagent (1 μl) was added to a mixture of 5 μl of serum and 195 μl LCAT assay buffer (150 mM NaCl, 10 mM Tris, 1 mM EDTA, 4 mM 2-mercaptoethanol, pH 7.4). The mixture (LCAT substrate reagent mixed with serum in LCAT assay buffer) was incubated for 30 min at 37 °C. Then, the reaction was stopped by adding the reconstituted READ reagent buffer (READ reagent, 150 mM NaCl, 10 mM Tris, 1 mM EDTA, pH 7.4). The mixture was transferred to a black 96-well microtiter plate and analyzed at excitation wavelength of 340 nm and emission wavelengths of 390 nm (hydrolyzed substrate) and 470 nm (non-hydrolyzed substrate) according to the manufacturer’s instructions (LCAT activity assay kit, Roar Biomedical, Inc., Calverton, NY, USA). The LCAT activity was determined by the ratio of the two emission intensities (390/470 nm).

### Cholesterol and cholesteryl esters quantitation

Quantitation of cholesterol and cholesteryl esters was done using colorimetric Cholesterol/Cholesteryl Ester Quantitation kit (EMD Millipore, Darmstadt, Germany) according to the manufacturer’s instructions. Briefly, serum was diluted 20-fold in cholesterol assay buffer and mixed with cholesterol probe, enzyme mix, and cholesterol esterase to quantify total cholesterol (cholesterol and cholesteryl esters). Quantification of cholesterol alone was done the same, except the cholesterol esterase was omitted in the reaction. The intensity of the color (representing the level of total cholesterol and cholesterol) was measured by exposing samples to 575 nm wavelength. Concentration was calculated using colorimetric standard.

### Phospholipid transfer protein (PLTP) activity and protein level

PLTP activity was measured using a colorimetric PLTP activity assay kit (Roar Biomedical, Inc., Calverton, NY, USA) according to the manufacturer’s instructions. Briefly, the activity was measured by combining serum (3 μl) with a mixture of 3 μl donor particles in 44 μl 1X assay buffer (150 mM NaCl, 10 mM Tris, 2 mM EDTA, pH 7.4). The samples were transferred to a black 96-well microtiter plate and 50 μl acceptor particles were added to each sample. The samples were incubated for 20 min at 37 °C, and then read at excitation wavelength of 465 nm and emission wavelength of 535 nm. To determine PLTP protein level, equal volume (μl) of serum was electrophoresed and probed using primary polyclonal rabbit PLTP antibody (ab20453, 1/1,000, Abcam, Cambridge, MA, USA) and secondary HRP-linked anti-rabbit IgG antibody (7074S, 1/5,000, Cell Signaling Technology, Inc., Danvers, MA, USA).

### Quantitative polymerase chain reaction (qPCR) for Apoa1

Livers were collected from 8-week-old male mice. Prior to the tissue collection, males were housed individually for 4 days, fasted for 4 h (7 am to 11 am) on the day of tissue collection, sacrificed by cervical dislocation, and perfused using PBS. Total RNA from whole liver was extracted using a Trizol Plus RNA Purification kit (Invitrogen Life Technologies, Grand Island, NY, USA). Complementary DNA (cDNA) was synthesized using an Omniscript Reverse Transcription kit (Qiagen, Valencia, CA) and used for qPCR with SYBR green (Applied Biosystems, Inc., Foster City, CA, USA) and Apoa1 primers (Forward: 5′ CACATATATAGACCAGGGAAGAAG 3′ and Reverse: 5′ CTGAAGGGTGTGGGTGAC 3′ from Primerdesign Ltd., Southampton, UK). The Apoa1 expression level was normalized with β actin (Forward: 5′ GCTTCTTT-GCAGCTCCTTCG 3′ and Reverse: 5′ CCCACGATGGAGGGGAATAC 3′ from Primerdesign Ltd., Southampton, UK). The relative expression difference was calculated using LinRegPCR (v11.0) [29] and Relative Expression Software Tool (REST©) [30].

### Statistics

All data represent the mean ± standard error of the mean (SEM) from the number of animals of each group, unless specified otherwise. Comparing means of more than two groups was performed using one-way ANOVA, followed by Tukey’s post-hoc test for multiple comparisons between specific groups. For comparisons of 2 groups, the non-parametric Mann–Whitney test was used to calculate levels of significance. Calculations of significance were done using Prism 8 (GraphPad Software, San Diego, CA). Level of statistical significance: *P < 0.05, **P < 0.01.

## Data Availability

All data generated or analyzed during the current study were included in this published article.

## Abbreviations

PCSK1: Proprotein convertase subtilisin/kexin type 1;
HDL: High-density lipoprotein
HDL-C: High-density lipoprotein cholesterol
APO1: Apolipoprotein A1
LCAT: Lecithin-cholesterol acyltransferase
PLTP: Phospholipid transfer protein
VLDL: Very low-density lipoprotein
LDL: Low-density lipoprotein
IDL: Intermediate-density lipoprotein
RT: Room temperature
WB: Western blot
SDS-PAGE: Sodium dodecyl sulphate–polyacrylamide gel electrophoresis
IEF: Iso-electro focusing
qPCR: Quantitative polymerase chain reaction
QTL: Quantitative trait loci
SEM: Standard error of the mean
ANOVA: Analysis of variance
SNV: Single nucleotide variant
SNP: Single nucleotide polymorphism
ABCA1: ATP-binding cassette subfamily member 1 transporter
ABCG1: ATP-binding cassette subfamily G member 1
SRB1: Scavenger receptor class B member 1
